# Unconjugated *p*-cresol activates macrophage macropinocytosis leading to increased LDL uptake

**DOI:** 10.1172/jci.insight.144410

**Published:** 2021-06-08

**Authors:** Lee D. Chaves, Sham Abyad, Amanda M. Honan, Mark A. Bryniarski, Daniel I. McSkimming, Corrine M. Stahura, Steven C. Wells, Donna M. Ruszaj, Marilyn E. Morris, Richard J. Quigg, Rabi Yacoub

**Affiliations:** 1 Department of Medicine, Division of Nephrology, Jacobs School of Medicine and Biomedical Sciences, and; 2 Department of Pharmaceutical Sciences, School of Pharmacy and Pharmaceutical Sciences, University at Buffalo, Buffalo, New York, USA.; 3 Department of Medicine, Bioinformatics and Computational Biology Core, Morsani College of Medicine, University of South Florida, Tampa, Florida, USA.

**Keywords:** Cardiology, Cell Biology, Apoptosis, Atherosclerosis, Macrophages

## Abstract

Patients with chronic kidney disease (CKD) and end-stage renal disease suffer from increased cardiovascular events and cardiac mortality. Prior studies have demonstrated that a portion of this enhanced risk can be attributed to the accumulation of microbiota-derived toxic metabolites, with most studies focusing on the sulfonated form of *p*-cresol (PCS). However, unconjugated *p-*cresol (uPC) itself was never assessed due to rapid and extensive first-pass metabolism that results in negligible serum concentrations of uPC. These reports thus failed to consider the host exposure to uPC prior to hepatic metabolism. In the current study, not only did we measure the effect of altering the intestinal microbiota on lipid accumulation in coronary arteries, but we also examined macrophage lipid uptake and handling pathways in response to uPC. We found that atherosclerosis-prone mice fed a high-fat diet exhibited significantly higher coronary artery lipid deposits upon receiving fecal material from CKD mice. Furthermore, treatment with uPC increased total cholesterol, triglycerides, and hepatic and aortic fatty deposits in non-CKD mice. Studies employing an in vitro macrophage model demonstrated that uPC exposure increased apoptosis whereas PCS did not. Additionally, uPC exhibited higher potency than PCS to stimulate LDL uptake and only uPC induced endocytosis- and pinocytosis-related genes. Pharmacological inhibition of varying cholesterol influx and efflux systems indicated that uPC increased macrophage LDL uptake by activating macropinocytosis. Overall, these findings indicate that uPC itself had a distinct effect on macrophage biology that might have contributed to increased cardiovascular risk in patients with CKD.

## Introduction

Interventions aimed at controlling traditional cardiovascular disease (CVD) risk factors in patients with chronic kidney disease (CKD) have proven less effective despite of treatment advances for hypertension, diabetes, and dyslipidemia (1–4). Notwithstanding aggressive therapies, patients with advanced CKD and end-stage renal disease (ESRD) on dialysis continue to suffer from a high annual mortality rate that reaches up to 20%–25%; more than half of these patients will die of cardiovascular causes (5–8). Given that approximately 15% of the adult American population currently lives with some level of CKD (9), and the close interrelations between CKD and CVD, it is imperative to investigate CKD-associated nontraditional CVD risk factors in an attempt to develop new therapeutic interventions. In general, nontraditional risk factors, such as inflammation, oxidative stress, and endothelial dysfunction, have been shown to play a key part in the development and outcome of CVD (10–13). One such component is CKD-associated dysbiosis (CKD-AD), which is the dysfunction within the microbiome-host relationship brought on by numerous factors in CKD (14, 15). There is an ever-expanding research base supporting an altered microbiome in CKD, and numerous studies have delineated several pathways whereby CKD progression promotes microbiome dysbiosis and vice versa (8, 16–25).

Microbiota dysbiosis itself can worsen traditional and nontraditional CVD risk factors. For example, fecal transplantation (FTX) from hypertensive individuals into germ-free mice elevated their blood pressure (26). Additionally, the permeability of the gastrointestinal (GI) tract wall can increase in CKD (27). This facilitates the enhanced absorption of bacterially derived metabolites and the translocation of gut bacteria, which have both been associated with increased CVD risk (27–32). A major contributor to the CKD-related increase in CVD risk is the elevation of adverse microbial metabolites in patients with renal diseases (23). It is hypothesized that CKD-AD will amplify the production of toxic compounds, the renal clearance of which is decreased in CKD. One example is *p*-cresol sulfate (PCS) (33). Whereas decreased renal function is linearly associated with decreased PCS clearance (34), no clear evidence supporting increased unconjugated *p*-cresol (uPC) and PCS production in response to CKD-AD is present. Tyrosine and phenylalanine colonic fermentation will generate uPC, which is converted into PCS predominantly by sulfotransferase 1A1 (SULT1A1) within hepatocytes, monocytes, and, to a limited extent, the intestinal epithelial cells (35). Increased levels of PCS are associated with poor renal, cardiac, and survival outcomes in patients with CKD and ESRD (36–38). PCS has been directly related to vascular endothelial injury (39), hepatotoxicity (40), and atherosclerosis severity (41). However, the toxicology and effects on lipid metabolism of its precursor uPC remain poorly defined. It has been long debated whether uPC carries any significant biological effects because earlier studies have failed to detect uPC in healthy subjects (42). However, De Loor et al. (43) report that up to 2 μg/mL is detected in patients on dialysis.

Evidence suggests that PCS is attributed to CVD risk through atherosclerotic plaque instability (44, 45) and increasing oxidative burst and macrophage phagocytosis (46). However, this limited body of evidence lacks in-depth analysis of macrophage cholesterol uptake machinery and, in most cases, is limited to epidemiological data. Moreover, the role of uPC and PCS in macrophage cholesterol uptake and retention and foam cell formation has yet to be studied. This is of great importance because it is the cornerstone of the formation of the atheroma and the development of CVD. We present here evidence that CKD-AD contributed to increased lipid deposits in the coronary arteries and mechanistically deciphered the proatherosclerotic role of uPC via altering both hepatic and macrophage lipid metabolism, resulting in dyslipidemia and the formation of lipid laden macrophages.

## Results

### CKD-AD increased coronary artery lipid deposits.

**Figure 1:**
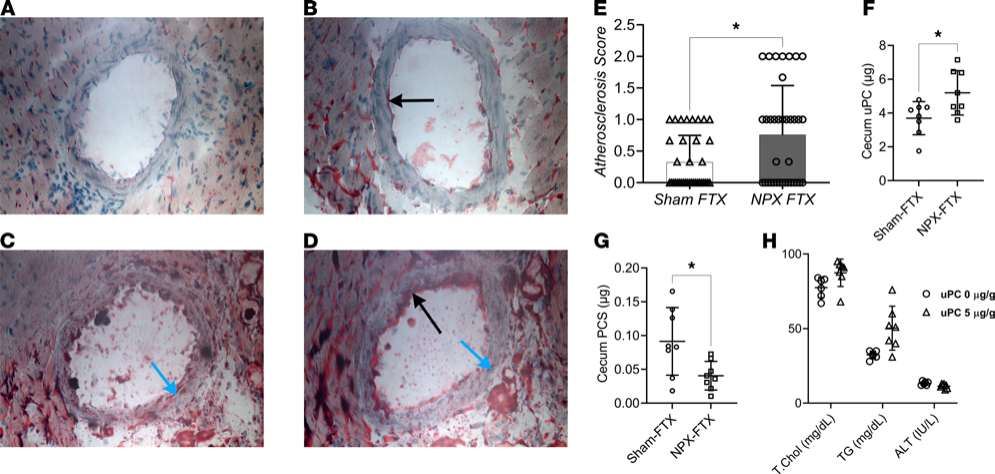
Fecal transplantation effects on coronary arteries’ lipid deposits, and lipid profile in response to unconjugated *p*-cresol exposure. (**A–D**) Oil Red O staining of the coronary arteries, scoring system representative images. Black arrows point at the subendothelial lipid deposits, blue arrows point at transmural lipid deposits. (**A**: score = 0; **B**: score = 1; **C** and **D**: score = 2.) (**E**) *ApoE*^–/–^ mice receiving fecal transplantation (FTX) from mice subjected to 5/6 nephrectomy (NPX) show a higher lipid deposits staining in the coronary arteries when compared with mice receiving FTX from sham-operated mice (*n* = 36 slide/group [4–6 slides/mouse]). (**F**) Cecum total *p*-cresol (uPC) content after FTX (*n* = 8/group). (**G**) Cecum total *p*-cresol sulfate (PCS) after FTX (*n* = 8/group). (**H**) Total cholesterol (T.Chol), triglycerides (TG), and alanine aminotransferase (ALT) levels after 2 weeks of i.p. injection with uPC or vehicle (*n* = 6/group). A significant increase in T.Chol and TG (Mann-Whitney *U*, *P* < 0.05) is noted in the uPC compared with control. Mann-Whitney *U*, *P < 0.05.

We and others have shown that CKD-AD is due to a variety of factors, including the underlying cause of renal dysfunction (47, 48) and therapeutic interventions common in patients with CKD (49–53). Here, we aimed to investigate the causality between CKD-AD and increased CVD risk by administering CKD fecal material to atherosclerosis-prone apolipoprotein E–knockout (*ApoE^–/–^*) mice (54) to determine if this would result in worsened coronary atherosclerotic lesions. Previously, we compared shifts in the colonic and small intestinal microbiomes between mice that underwent 5/6 nephrectomy (NPX) and sham-operated controls (25). Cecum material from those mice were used as FTX donors in the current experiment (detailed information in regards to the fecal material taxonomic analysis, renal function, and experimental procedures have been previously published; ref. 25). After a 3-day gut sterilization using broad-spectrum antibiotics, fecal material from NPX and sham-operated mice were transplanted 3 times weekly into *ApoE^–/–^* mice receiving a high-fat diet (HFD) for a total of 19 weeks. No significant differences were noted in weight after FTX between the 2 groups (Supplemental Figure 1; supplemental material available online with this article; https://doi.org/10.1172/jci.insight.144410DS1). Coronary artery lipid deposits were then compared. Oil Red O staining was performed on frozen cardiac sections at the level of papillary muscles. A scoring system was established to evaluate lipid deposits with representative images depicted in Figure 1, A–D. Sections were blindly evaluated in triplicates. *ApoE^–/–^* mice receiving FTX from NPX mice showed increased lipid deposits when compared with those receiving FTX from sham-operated mice, supporting the role of CKD-AD in CVD (Figure 1E). To ensure successful FTX, we performed 16S ribosomal DNA sequencing on stool palettes obtained 1 day before euthanasia (Supplemental Figure 2). Findings revealed group clustering based on the FTX donor, indicating a successful FTX procedure.

We then sought to evaluate whether changes noted in the lipid deposits after FTX can be attributed to a dysbiosis-related pathway. We performed Kyoto Encyclopedia of Genes and Genomes (KEGG) and Phylogenetic Investigation of Communities by Reconstruction of Unobserved States (PICRUSt) analysis (55) of the stool bacteria from *ApoE^–/–^* mice receiving FTX from either NPX or sham-operated donors (Supplemental Figure 3). Analysis revealed that mice receiving FTX from NPX donors exhibited a microbiome profile compatible with an increased *p*-cresol formation pathway and a decreased phenol degradation pathway. These findings led us to investigate the effects that CKD-AD has on uPC production. Cecum material from *ApoE^–/–^* mice receiving FTX from NPX donors exhibited higher total uPC when compared with those receiving FTX from sham-operated donors (Figure 1F), supporting the hypothesis that CKD-AD results in increased uPC production. Additionally, cecum PCS was higher in mice receiving FTX from sham-operated donors when compared with those receiving FTX from NPX donors (Figure 1G). In-depth KEGG and PICRUSt analyses revealed aberrancy in multiple metabolism pathways (Supplemental Figure 4 and Supplemental Table 1. Of importance to atherosclerosis risk, the fecal microbiome profile of *ApoE^–/–^* mice receiving FTX from NPX donors exhibited aberrant lipid, carbohydrate, amino acid, and xenobiotic metabolism pathways (Supplemental Figure 4). The increased uPC production, abnormal lipid deposits in the coronary arteries, and altered metabolism pathways supported a role for CKD-AD in CVD risk.

### uPC increased total cholesterol and triglyceride levels.

After establishing increased coronary artery lipid droplets in mice receiving FTX from NPX, we sought to evaluate the mechanisms by which CKD-AD might influence CVD risk. Although limited epidemiological and experimental animal studies suggest that PCS might be the culprit (36–38, 44, 45), we evaluated the biological effects of its precursor uPC. Due to the lack of information on the effect of uPC on CVD risk, we conducted daily i.p. injections of uPC (5 μg/g weight) or vehicle for 2 weeks. We chose i.p. for the route of administration to mimic the physiologic path uPC undergoes: i.p. uPC passes unchanged through the peritoneal membrane, enters the mesenteric veins, travels to the portal vein, and then undergoes hepatic first-pass metabolism (56). Mice receiving uPC exhibited significantly higher total cholesterol and triglyceride serum concentrations without an elevation of alanine transaminase when compared with vehicle controls (Figure 1H). This indicated uPC altered lipid metabolism in vivo.

### uPC induced hepatic and aortic wall lipid deposits.

**Figure 2:**
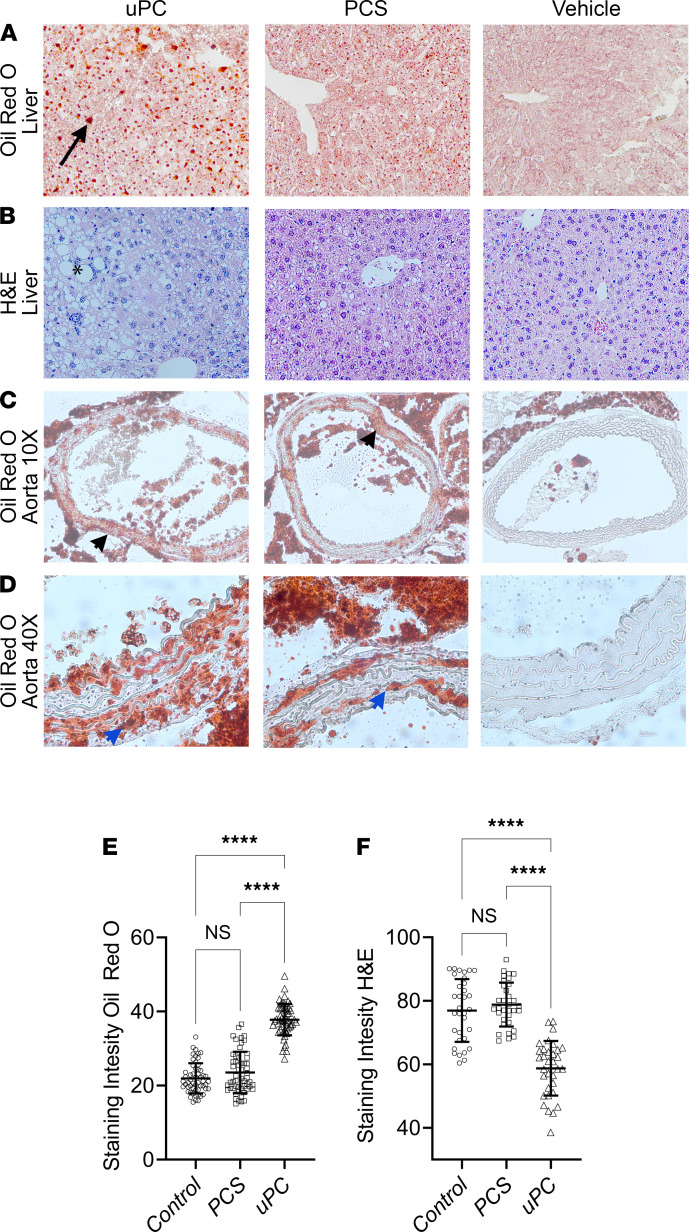
Hepatic and aortic wall lipid accumulation in response to unconjugated *p*-cresol and *p*-cresol sulfate exposure. Mice receiving daily i.p. injections of *p*-cresol (uPC) for 2 weeks showed increased hepatic lipid retention (Oil Red O staining, arrow) when compared with those receiving either *p*-cresol sulfate (PCS) or vehicle (**A** and **E**). H&E staining shows a significant fatty necrosis (*) in the liver in those receiving uPC (**B** and **F**). Both uPC and PCS resulted in increased lipid depositions in the aortic walls (black arrowhead) when compared with vehicle (**C**). However, uPC administration resulted in a more extensive and diffuse deposits (blue arrowhead) (**D**). (**A**–**D**: *n* = 6 mice/group; **E** and **F**: *n* = 6–9 slides/mouse.) Tukey’s test, ****P < 0.0001.

Because we did not measure the serum or portal vein concentrations of uPC and PCS, the effects uPC exhibited on lipid metabolism could have been attributed to increased circulating PCS secondary to increased levels of its substrate. To address this facet, C57BL/6J male mice fed HFD received daily i.p. injections of uPC, PCS, or vehicle for 2 weeks. Mice that received uPC had extensive hepatic fatty changes, as indicated by Oil Red O staining when compared with the other groups (Figure 2, A and E). H&E staining revealed heightened hepatic fatty necrosis in the uPC group when compared with both PCS and vehicle groups (Figure 2, B and F). We also evaluated a transverse section of the ascending thoracic aorta in these 3 groups (Figure 2, C and D). Both PCS and uPC resulted in increased fat deposition in the aortic wall when compared with vehicle controls. However, mice receiving uPC exhibited diffuse and extensive fatty deposits when compared with PCS. We then sought to evaluate if hepatic fatty changes persisted if mice were not fed HFD. C57BL/6J male mice fed normal laboratory rodent chow, received daily i.p. injections of uPC, PCS, or vehicle for 2 weeks. No significant differences were noted between the 3 groups regarding hepatic fatty changes (Supplemental Figure 5). This indicated that uPC required a HFD to induce hepatic fatty changes and that lipid-induced hepatic toxicity was aggravated in response to uPC rather than directly induced.

### uPC exhibited higher potency than PCS to increase macrophage LDL uptake and retention.

**Figure 3:**
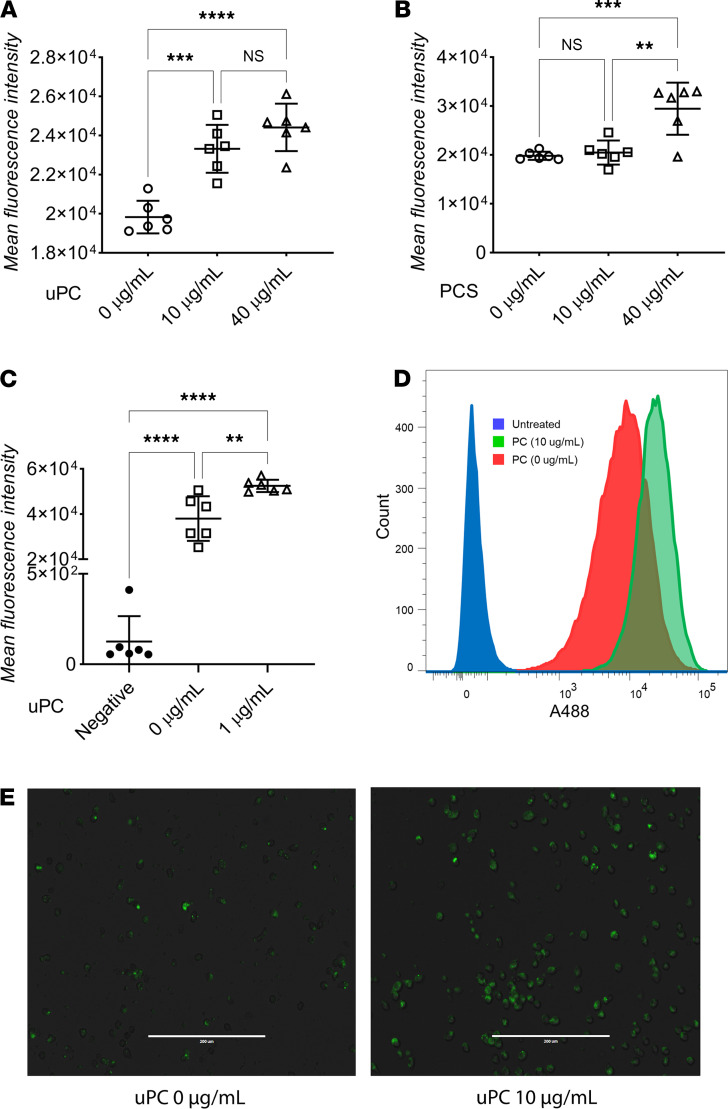
A488-labeled LDL uptake in RAW cells in response to unconjugated *p*-cresol and *p*-cresol sulfate exposure. (**A** and **B**) MFI of RAW cells treated with A488 LDL (1 μg/mL) and different concentrations of *p*-cresol (uPC) and *p*-cresol sulfate (PCS) show increased LD uptake with uPC, whereas lower PCS concentrations did not. (**C** and **D**) MFI of RAW cells treated with 0 or 1 μg/mL uPC (negative = no A488 LDL, no uPC). (**C**) Significantly increased LDL uptake and retention after uPC treatment. (**D**) Representative flow cytometry MFI analysis. (**E**) Immunofluorescence representative images of A488-labeled LDL RAW cells uptake after exposure to uPC. (**A**–**D**: *n* = 6/group; **E**: *n* = 3/group.) Tukey’s test, **P < 0.01, ***P < 0.001, ****P < 0.0001.

Next, we conducted studies to understand the mechanisms contributing to the difference in aortic lipid deposits between uPC and PCS. After an overnight serum starve, the murine immortalized macrophage cells RAW 264.7 (RAW) were incubated with varying concentrations of uPC and PCS along with Alexa Fluor 488–labeled LDL (A488 LDL) for 24 hours (Figure 3, A and B). We chose 0, 10, and 40 μg/mL for both metabolites because these concentrations corresponded with the concentrations in healthy individuals (~0.5 μg/mL) and patients with moderate CKD (~10 μg/mL) and advanced CKD (~40 μg/mL (57–61). Incubating RAW cells with PCS induced LDL retention only at 40 μg/mL, whereas uPC induced significant LDL retention at 10 and 40 μg/mL. Studies have shown peripheral uPC concentrations in patients on dialysis can approach 2 μg/mL (43). To establish the effects of uPC on macrophage LDL uptake in biologically relevant concentrations, we observed that uPC increased LDL retention at a concentration of 1 μg/mL (Figure 3, C and D). This was supported by fluorescent imaging, which showed increased A488 LDL uptake after uPC treatment (Figure 3E). These results indicate that uPC exhibited an ability to stimulate the development of aberrant cholesterol handling in a macrophage in vitro. As macrophages express SULT1A1 (62–64), they possess the ability to generate PCS from uPC. Thus, one could question whether changes seen in uPC are attributable to the formation of PCS. However, PCS itself did not induce LDL retention until high concentrations (40 μg/mL), whereas uPC induced LDL retention at much lower concentrations (1 μg/mL), indicating that the biological effects seen in the current study were due to uPC itself.

### uPC, but not PCS, reduced macrophage viability and increased apoptosis.

**Figure 4:**
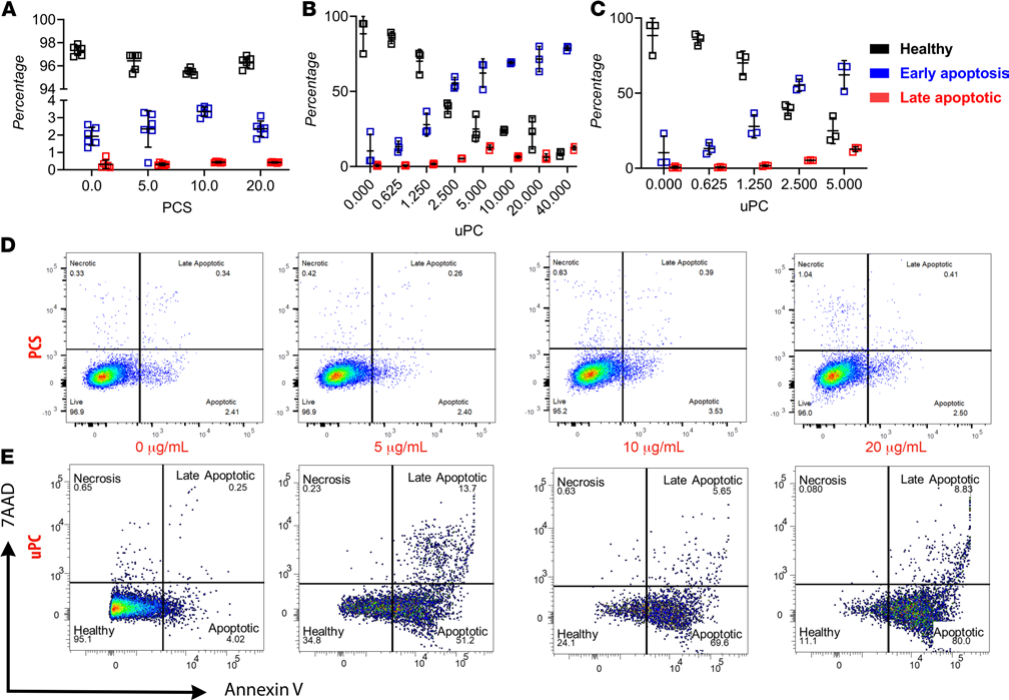
RAW cell apoptosis in response to both unconjugated *p*-cresol and *p*-cresol sulfate exposure. Although *p*-cresol sulfate (PCS) did not result in increased apoptosis (**A**), *p*-cresol (uPC) showed a significant dose dependent increase in apoptosis (**B**). These changes were noted in doses as low as 1–2 μg/mL (**C**), a concentration previously considered to be too low to have any biological effects. Representative images of the flow cytometric analysis illustrating the dose dependent increase in apoptosis in response to uPC exposure (**E**), and not PCS (**D**). *n* = 6 each treatment/time point in **A–E**. 7AAD, 7-amino-actinomycin D.

In atherosclerotic plaques, activated macrophages contribute to the progression and worsening of the lesion by releasing inflammatory cytokines (65). The poor clearance of apoptotic and necrotic macrophages from the plaque will result in the formation of the lipid core, which accelerates plaque development and instability (66, 67). Therefore, we conducted in vitro work to provide greater insight on how uPC and PCS affects macrophage viability and apoptosis (Figure 4, A–E). Although no significant changes were noted when different concentrations of PCS were used (Figure 4, A and D), flow cytometry analysis demonstrated uPC dose-dependently increased RAW cell apoptosis and necrosis (Figure 4, B, C, and E). It is important to note that early apoptosis in RAW cells occurred at approximately 1 μg/mL of uPC (Figure 4C), a concentration previously deemed to be too low and biologically irrelevant in patients on dialysis. Coupled with the knowledge that patients on dialysis have as high as 2 μg/mL of circulating uPC (43), our findings support our hypothesis that uPC induced macrophage LDL retention and apoptosis and carried biologically relevant consequences in renal patients. These processes are the cornerstone in the development and progression of atherosclerotic plaque in CVD.

### Cholesterol influx and efflux pathways in response to uPC and PCS.

Macrophages take up vast amounts of modified apoB-containing lipoproteins to become “foam cells” (68, 69). This decreases their mobility and results in an inflammatory response within the vessel wall (69). Given this, we sought to first evaluate if changes in LDL retention after uPC and PCS treatments (CF Figure 3, A and B) were due to an increased uptake capacity (influx). We characterized the major pathways associated with LDL uptake in RAW cells: receptor-mediated endocytosis and receptor-independent pinocytosis (70–73).

**Figure 5:**
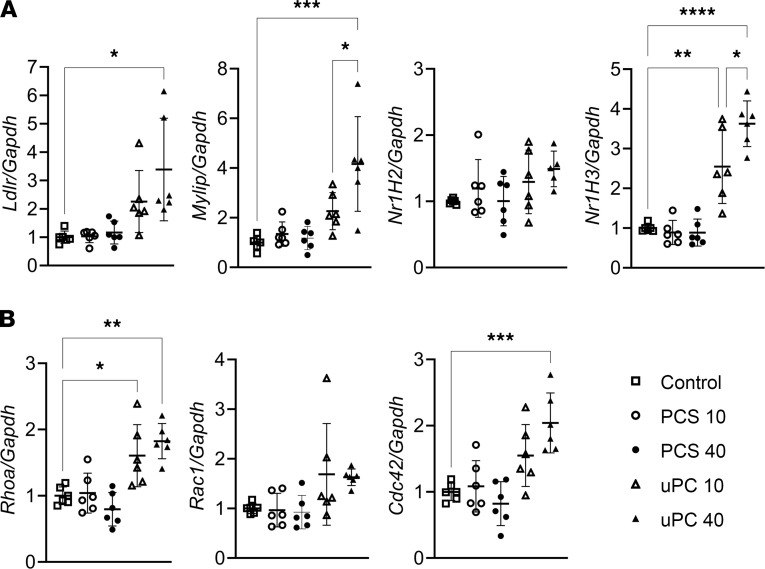
Endocytic pathways gene expression in response to unconjugated *p*-cresol and *p*-cresol sulfate exposure. Relative expression of receptor-mediated endocytosis (**A**) and receptor-independent pinocytosis (**B**) genes in RAW cells in response to different concentrations of *p*-cresol (uPC) and *p*-cresol sulfate (PCS). PCS doses were not associated with any significant change in the relative expression of both endocytosis and pinocytosis genes. In comparison, a dose-dependent increase in LDL endocytosis and pinocytosis genes are noted in response to uPC exposure. (**A** and **B**: *n* = 6/group.) Tukey’s test, *P < 0.05, **P < 0.01, ***P < 0.001, ****P < 0.0001.

No changes were observed in LDL endocytosis or pinocytosis genes after PCS administration (Figure 5, A and B). This indicated that PCS did not affect LDL macrophage uptake through either endocytosis or pinocytosis. On the contrary, uPC administration resulted in an induction of the LDL receptor mRNA (*ldlr*) together with increased expression of nuclear receptor subfamily 1 group H members (*Nr1H*) 3 and myosin regulatory light chain interacting protein (*Mylip*, also known as inducible degrader of the LDL-receptor [*Idol*]). Furthermore, only uPC-treated RAW cells displayed an increase in genes associated with liquid phase receptor-independent pinocytosis, a major LDL uptake pathway in macrophages (Figure 5, A and B) (73, 74). These findings further support the role of uPC in this endocytic pathway.

However, cholesterol efflux via the reverse cholesterol transport (RCT) pathway can revert this phenotype, leading to macrophage egress from lesions and a subsequent reduction in lesion burden (75). Thus, LDL retention in response to uPC exposure could be due to modifications in the RCT system. We addressed this by analyzing the RCT pathway in uPC/PCS-treated RAW cells. Our experiments revealed a trend toward increased relative expression of *Shp* (a major regulator of the RCT pathway), decreased expression of *Cyp7a1* with 10 μg/mL uPC, and an induction in *Cyp7a1* after PCS (Supplemental Figure 6). This shows the RCT is not likely the mechanism by which uPC increased LDL retention. However, due to the high C^t^ on PCR, we could not confirm whether uPC significantly affected the RCT pathway.

### uPC increased LDL uptake through activating micropinocytosis.

**Figure 6:**
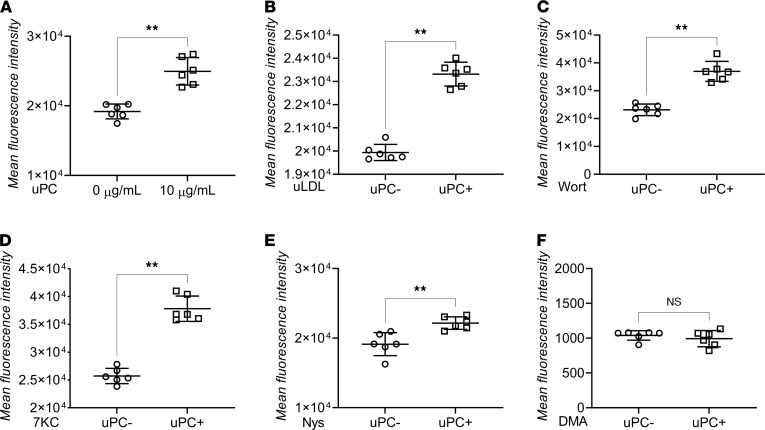
A488 LDL uptake in response to unconjugated *p*-cresol exposure under chemical inhibition of different uptake pathways. (**A**) RAW cells A488 LDL uptake without chemical inhibition showing increased uptake after exposure to *p*-cresol (uPC). (**B**) RAW cells A488 LDL uptake after inhibiting receptor-mediated endocytosis using 200× unlabeled LDL (uLDL) had no effect on the uPC induced increased LDL uptake. (**C**) RAW cells A488 LDL uptake after inhibiting phagocytosis using wortmannin (Wort) had no effect on the uPC induced increased LDL uptake. (**D**) RAW cells A488 LDL uptake after inhibiting reverse cholesterol transport system using 7 ketocholesterol (7KC) had no effect on the uPC-induced increased LDL uptake. (**E**) RAW cells A488 LDL uptake after inhibiting micropinocytosis using nystatin (Nys) had no effect on the uPC-induced increased LDL uptake. (**F**) RAW cells A488 LDL uptake after inhibiting macropinocytosis using dimethyl amiloride (DMA) showing complete blockage of uPC induced increased LDL uptake. These results indicate that macropinocytosis is the site of action where uPC increases macrophage LDL uptake. 1 μg/mL of A488 LDL and 10 μg/mL uPC were used in all experiments. (**A–F:** *n* = 6/group.) Mann-Whitney *U*, **P < 0.01. ns, not significant.

Studies have shown that LDL and lipid entry to macrophages is mostly governed by liquid phase pinocytosis rather than receptor-mediated LDL uptake (73, 74) or the RCT system (76–79). To be specific, phagocytosis and macropinocytosis are the 2 major pathways for macrophage LDL uptake (70, 73). Basal *Cyp7a1* (RCT system) expression in RAW cells is low (76), indicating a possibly limited RCT pathway role in macrophage LDL uptake. We first confirmed this by performing competitive inhibition using 200× unlabeled LDL (uLDL; Supplemental Figure 7). Increased A488 LDL concentrations resulted in increased uptake regardless of receptor saturation (200× uLDL), confirming that liquid phase pinocytosis rather than receptor-mediated endocytosis is the main mechanism of LDL entry into macrophages. We then aimed to further evaluate the exact mechanism by which uPC increases LDL uptake by pharmacologically inhibiting different influx and efflux pathways in RAW cells exposed to uPC. These pathways included the RCT system (using 7 ketocholesterol [7KC]), receptor-mediated endocytosis (using competitive inhibition, 200× uLDL), phagocytosis (using wortmannin), micropinocytosis (using nystatin [Nys]), and macropinocytosis (using dimethyl amiloride [DMA]). Because the incubation duration with chemical inhibitors and A488 LDL is only 1 hour in this experiment, in comparison with 24 hours shown in Figure 3, we first sought to evaluate if this duration is enough to replicate similar increased LDL uptake after uPC exposure (Figure 6A [10 μg/mL uPC] and Supplemental Figure 8 [1 μg/nL uPC]). After confirming, we performed the pharmacological inhibition experiments. As noted in Figure 6, B–F, uPC still significantly induced an increased A488 LDL uptake in response to all inhibitors except DMA, indicating that macropinocytosis activation was the pathway by which uPC increased macrophage LDL uptake.

### uPC increased F-actin richness and filopodia length.

**Figure 7:**
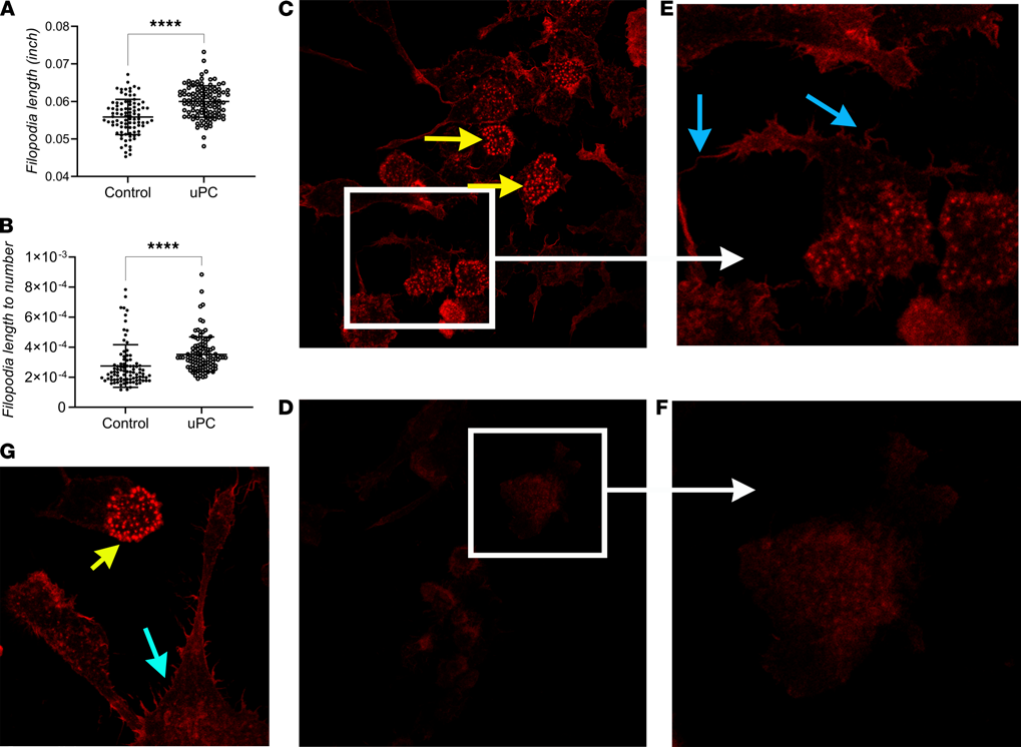
Filopodia length and actin cytoskeleton arrangement in response to uPC exposure. uPC exposure resulted in increased filopodia length (**A**) when compared with control. Adjusting filopodia length to the number of filopodia present in each slide (**B**) revealed similar results. F-actin staining using phalloidin-TRITC indicates increased F-actin after exposure to uPC (**C**) when compared with control (**D**). High-power magnification of RAW cells reveal increased filopodial actin (blue arrow) in response to uPC (**E** and **G**) when compared with control (**F**), along with formation of actin-rich structures (yellow arrow). These structures localized at the cell-slide region. (**A** and **B**: *n* = 6–8 random images/slide; **C**–**F**: *n* = 12 slides/group.) Mann-Whitney *U*, ****P < 0.0001.

To build upon the findings that uPC increased LDL uptake by activating macropinocytosis, we investigated whether exposure to uPC increases filopodia length and number and affects actin cytoskeleton arrangement. Filopodia are protrusions that arise from the plasma membrane and contain actin filaments (80). These projections form an investigatory system seeking to endocytose extracellular small particles and fluid content in a nonspecific manner. Macropinocytosis is mainly an actin-dependent process. It consists of multiple processes of actin cytoskeleton–driven ruffle formation, closure of the ruffle, and dissociation of actin filaments from the ruffle to form macropinosomes (81). Actin filaments in the filopodia form a bundle shape that arises from the actin network (81, 82). RAW cells exposed to uPC exhibited increased filopodia length when compared with control (Figure 7). F-actin staining using phalloidin-TRITC revealed intense signal in RAW cells exposed to uPC when compared with control (Figure 7). High-resolution magnification indicated increased filopodial actin staining in response to uPC, along with the formation of actin rich structures. These structures localize at the slide-cell junction. Altogether, these findings add another layer of evidence that uPC exposure increased macrophage LDL uptake through activating macropinocytosis.

## Discussion

In this study, we demonstrate that CKD-AD accelerated lipid deposits in coronary arteries of *ApoE^–/–^* mice fed a HFD, further supporting the role CKD-AD has in the development and risk of CVD. Whereas prior epidemiological and, to a limited degree, experimental animal studies indicated that PCS is associated with this risk (36–41, 44–46), we present here an in-depth mechanistic analysis of how its precursor uPC contributed to the development of CVD through characterizing its effect on macrophage viability and their ability to uptake and retain LDL. We further demonstrated the enhanced hepatic lipid accumulation in response to uPC exposure. This report does not discredit the dyslipidemic effects of PCS; rather, it presents evidence that uPC carries a significant role in dyslipidemia and CVD predisposition. This knowledge further strengthens the need for therapeutic interventions aimed at decreasing intestinal uPC concentrations that limit its absorption to alleviate the hepatic burden, decrease foam cell formation, and ultimately decrease CVD risk.

CKD-AD was hypothesized to increase the production of toxic compounds whose renal clearances are decreased in CKD (33). Here, we present an early evidence supporting this hypothesis (Figure 1F and Supplemental Figure 3). Additionally, the increased intestinal permeability noted in CKD (i.e., “leaky gut”) (27) will facilitate and enhance absorption of these bacterially derived metabolites as noted in both patients with CKD and experimental animal studies (83–92). uPC generated by the gut flora is absorbed by intestinal epithelial cells and delivered to the liver for detoxification via the portal vein. Because sulfonated products are not absorbable in the intestinal tract (93–99), only uPC can be absorbed. uPC will then undergo extensive first-pass metabolism, such that peripheral serum concentrations of uPC are negligible in healthy individuals (93–104). This implies that the liver faces the brunt of the uPC load and detoxification burden. Sulfonation is an important pathway in the biotransformation of a number of endogenous and xenobiotic substrates such as phenols (e.g., uPC) (105). This process is mediated by aryl sulfotransferase and is the basic pathway of forming PCS from uPC. There is an extensive body of evidence supporting the detrimental effects ESRD has on dyslipidemia (106–111) and the development of nonalcoholic steatohepatitis (NASH) and nonalcoholic fatty liver disease (NAFLD) (112–117). This coupled with the findings of downregulated hepatic sulfotransferase expression and activity in both NASH and NAFLD (105, 118) could possibly explain the increased (detected) uPC concentrations in patients on dialysis as opposed to healthy individuals.

Macrophage cholesterol uptake and the formation of lipid-laden macrophages (foam cells) plays an essential role in the development of the atherosclerotic plaque and subsequently CVD (119). In atherosclerotic plaques, activated macrophages contribute to the progression and worsening of the lesion by releasing inflammatory cytokines (65). The poor clearance of apoptotic and necrotic macrophages in the plaque will result in the formation of the lipid core, which itself is an inflammatory stimulus to other macrophages, facilitating further damage and accelerating the progression of the plaque (66, 67). Our findings of increased RAW cell apoptosis in response to uPC at concentrations as low as 1 μg/mL suggest that uPC concentrations in uremic patients could have attributed to the development and possibly the severity of CVD through aggravating macrophage apoptosis, resulting in enhanced formation and progression of the lipid core. However, it is important to note that these in vitro results need further in vivo confirmation.

Basal *Cyp7a1* (RCT system) expression in RAW cells is low (76), and recent data suggest that it might play a role in response to cholesterol under stress conditions (77, 78). *Cyp7a1* stable overexpression in RAW cells decreases LDL accumulation (76), and increasing its activity in macrophages reduces cholesterol accumulation and LDL toxicity (79). Our results indicate that uPC exposure resulted in a trend toward increased *Shp* and decreased *Cyp7a1* expression, demonstrating a possible site of action in increasing LDL retention. However, the relative expression of both genes in RAW cells is low. When coupled with the findings that blocking the RCT system using 7KC still caused increased LDL uptake in macrophages in response to uPC, these results suggest this pathway was not the culprit. Exposure to PCS resulted in increased *Cyp7a1* expression. Whether this would result in activating the RCT system in response to PCS, thereby increasing cholesterol efflux, is unclear. However, our data show that high concentrations of PCS (40 μg/mL) resulted in increased rather than decreased LDL uptake. Although we did not measure uPC and PCS concentrations after exposure to each metabolite, these findings can be attributed to the limited desulfonation (by hydrolysis) of PCS used (42, 43, 120), resulting in the generation of uPC at low concentration and hence an increased LDL uptake. Similarly, a vast body of evidence suggests that the RCT system is not the major player in macrophage LDL uptake (70, 73, 74, 76–79).

The modality of pinocytosis is heavily governed by the molecule size (diameter). Phagocytosis consists of uptake of large molecules (>500 nm), such as microorganisms and cell debris (121), and macropinocytosis is responsible of uptake of molecules greater than 10 nm (70, 122–124). Although LDL particle populations are heterogeneous in size, they generally average 10–20 nm (125–127), further confirming the notion that LDL uptake is mainly governed by macropinocytosis (70). Our gene expression data highlights 3 possible mechanisms by which uPC increases LDL uptake. Increased expression of *Ldlr* (implicating receptor-mediated endocytosis), *Cdc42* (implicating phagocytosis), and *Rhoa* (implicating macropinocytosis) were noted after exposure to uPC. However, changes in gene profiles do not definitively establish the site of action for uPC-induced increased uptake of LDL. Thus, we chemically inhibited each pathway and evaluated RAW cell LDL uptake in response to uPC exposure. Inhibition of both receptor-mediated endocytosis and phagocytosis failed to block the increased LDL uptake. In contrast, macropinocytosis inhibition blocked uPC-induced increased LDL uptake, indicating macropinocytosis is the primary endocytic route. These findings were further supported by an increase in actin richness and filopodia length noted in response to uPC exposure. The exact, in-depth mechanism by which uPC activates macropinocytosis in RAW cells remains to be defined and was beyond the scope of the current study.

Although we present multiple levels of evidence suggesting that uPC rather than PCS increased macrophage apoptosis and LDL uptake, it is essential to highlight the fact that RAW cells are a macrophage-like cell line. RAW cells were originated from an Abelson leukemia virus–transformed cell line derived from BALB/c male mice and have been used for over 40 years to study macrophage biology. These cells have been described as an appropriate model of macrophages (128–130). However, further in vivo and ex vivo experiments utilizing primary macrophages to confirm our findings are warranted, because macrophage plasticity, polarization, and further differentiation might influence the observed effects (131–133).

In conclusion, our study implicates uPC in the process of CVD through multiple pathways. Although uPC itself increases macrophage’s ability to take up LDL and form lipid-laden macrophages, it also decreases its survival, which would ultimately play a role in atherosclerotic plaque formation and instability. It also affects the hepatic handling of lipids, leading to increased total cholesterol and triglycerides concentrations. Because sulfonated phenols are not intestinally absorbed, the increased PCS concentrations noted in the epidemiological studies might reflect increased uPC absorption and/or production. Thus, the PCS-related deleterious effects noted in the association studies might be partially explained by increased uPC concentrations. Although this indicates that PCS concentrations might act as surrogates to uPC (concentration, production, and absorption), further studies are warranted to reach definitive conclusion. Furthermore, mechanistic studies aimed at deciphering the exact molecular mechanisms by which uPC increases macrophage apoptosis and activates macropinocytosis are ongoing. These findings will undoubtedly enrich and improve our understanding of how uremic toxins increase CVD risk.

## Methods

### Mice.

Mice were housed under specific pathogen–free conditions at the University at Buffalo laboratory animal facilities. Mice received standard laboratory rodent chow or HFD (Teklad rodent diet TD.06414) ad libitum as detailed below. Distilled autoclaved water was provided in bottles. Lighting was on a 12-hours-on/12-hours-off cycle. Body weight was measured daily after interventions for 1 week and then every 4 weeks.

To generate *ApoE^–/–^* mice (B6.129P2-*Apoe^tm1Unc^*/J), heterozygote (*ApoE^+/-^*) breeding pairs were acquired from The Jackson Laboratory (stock 002052). To account for cage effects on the gut bacterial microbiota and ensure similar gut microbiota before FTX at baseline, we followed a standardized protocol. Homozygote (*ApoE^–/–^*) male littermates were weaned and cohoused in the same cage (*n* = 2–4 each cage) for 13 weeks (16 weeks of age) before gut sterilization. Afterward, mice were then single housed and provided HFD ad libitum and divided equally (*n* = 8/group) to receive fecal material obtained from the cecum(s) of CKD mouse model (NPX) or control (sham). Fecal material was obtained from mice used in previous experiments (25). FTX was conducted 3 times per week for 19 weeks. At the endpoint, mice were euthanized under isoflurane (2% with 1.5 L/min O_2_).

Eight-week-old C57BL/6J male mice were acquired from The Jackson Laboratory (stock 000644). For lipid profile experiments, mice were housed in groups (5 per cage, 1 cage per group) for 2 weeks and then administered i.p. injections of either vehicle or uPC (5 μg/g weight; MilliporeSigma, C85751) daily for 2 weeks (5 days a week). A similar approach was followed when evaluating hepatic and aortic lipid accumulation. Eight-week-old male mice were initially housed for 2 weeks (5 per cage, 1 cage per group). Mice then received HFD starting the day before the first i.p. injection. Equal volumes of vehicle, uPC (5 μg/g weight) or PCS (5 μg/g weight; MilliporeSigma, SMB00936) were administered via i.p. injections as described above for 2 weeks.

### FTX.

Here, we utilized fecal material obtained from our previously published CKD mice model and controls (25). In brief, 8-week-old C57BL/6J male mice (*n* = 10 /group) underwent NPX or sham operation. Nine weeks postoperatively, cecum material from sham and NPX groups was obtained during euthanasia, immediately snap-frozen in liquid nitrogen, and stored in –80°C (25). Prior to FTX, cecum materials were thawed on ice, homogenized in transfer buffer (1 mL prereduced sterile PBS containing 0.05% cysteine HCl on ice for each 120-mg cecum weight), vortexed at high speed for 1 minute, and then centrifuged at 800*g* twice for 2 minutes each. The supernatant was then stored at –80°C and used for FTX. Sixteen-week-old *ApoE^–/–^* male mice underwent gut sterilization as follows: streptomycin 1 mg/kg (MilliporeSigma, 51277) and ciprofloxacin 10 mg/kg (MilliporeSigma, 17850) via oral gavage once, followed by 3 days of metronidazole (10 mg/mL; MilliporeSigma, M1547) and ampicillin (10 mg/mL; MilliporeSigma, A9393) in drinking water for 1 week. Artificial sweeteners were mixed in the drinking water containing the antibiotics. After gut sterilization, mice were single housed. Fecal materials were given 3 times per week for 19 weeks (50 μl via oral inoculation). Cage bedding was changed daily during the time of gut sterilization and the first week of FTX to prevent mice from consuming the fecal pellets in the cage. After that, cage bedding was changed once weekly.

### Euthanasia and tissue harvesting.

At the endpoint of each experiment, mice were euthanized under isoflurane (2% with 1.5 L/min O_2_). After achieving proper anesthesia, abdominal and thoracic cavities were exposed and blood was collected via right ventricle cannulation. A left ventricle cannula was placed, then 50 mL of ice-cold PBS was infused over 5 minutes after disturbing the inferior vena cava (below the renal veins) for drainage. Liver sections and heart were either snap frozen or placed in *RNAlater* (Thermo Fisher, AM7021). Ascending thoracic aorta was placed in 5 mL ice-cold PBS with gentle shaking for 5 minutes on ice, then snap frozen. Samples were then stored in –80°C. One liver section was placed in 10% formalin for 24 hours followed by embedding and sectioning for histological analysis.

### Oil Red O staining and hematoxylin eosin staining.

Oil Red O staining was performed on frozen liver and aortic sections (10-μm thick) following the manufacturer’s instructions (MilliporeSigma, MAK194). A scoring system was devised to evaluate the extent of lipid deposits in the coronary arteries (Figure 1, A–D). Hearts were sectioned at the level of the papillary muscles and 6 slides per mouse were stained with Oil Red O. Each section was reviewed and scored blindly by 3 investigators. The 3 investigators’ average score of each slide was used for analysis. A score of 0 was given when no lipid deposits were noted in the subendothelial and transmural sections of the ascending thoracic aorta. Mild subendothelial lipid deposits were scored as 1, and extensive subendothelial and/or transmural lipid deposits were scored as 2. Paraffin embedding, sectioning, and H&E staining were performed at the Roswell Park Comprehensive Cancer Center histology core. Images were obtained using Leica DM6000 Microscope, with Nuance EX Multispectral Camera ×40.

### Quantitative PCR.

PCR was performed using SYBR Green Master Mix (Applied Biosystems) and the Applied Biosystems 7500 Real-time PCR system. Gene expression was normalized to *Gapdh* and fold change in expression relative to the control group was calculated using the 2^-ΔΔCt^ method. Primers used in PCR are listed in Supplemental Table 2.

### Cell culture.

RAW cells (ATCC TIB-71) were cultured in a 5% CO_2_, 95% O_2_ humidified incubator at 37°C. Cells were grown in DMEM supplemented with 10% heat-inactivated FBS that was changed every 2 days.

### LDL uptake.

Cells were seeded in 6-well culture plates at 30% confluency and grown for 24 hours, followed by overnight starvation. Cells were then incubated in different concentrations of uPC and PCS along with 1 μg/mL A 488 LDL (Invitrogen, L23380) in DMEM for 24 hours. Plates were washed with ice-cold PBS 3 times. LDL uptake was either directly visualized using an Olympus BX-60 fluorescent microscope with Zeiss Axiocam digital color camera or measured using a BD LSRFortessa flow cytometer. Several inhibitory agents were used to delineate the mechanisms underlying the effects uPC had on A488 LDL uptake. The RCT system was blocked using 7KC (5 μg/mL; MilliporeSigma, C2394); phagocytosis was blocked using wortmannin (100 nM; MilliporeSigma, W1628); macropinocytosis was blocked using DMA (200 μg/mL; MilliporeSigma, A4562); micropinocytosis was blocked using Nys (250 IU/mL; MilliporeSigma, M1638); and LDL receptor-mediated endocytosis was blocked using competitive inhibition (receptor saturation) via addition of 200 μg/mL uLDL (200×; MilliporeSigma, 437644). Cells were first incubated with or without uPC for 24 hours (while under starvation), followed by incubation with inhibitor(s) for 30 minutes, then with a cocktail of: inhibitor(s), uPC (0, or 10 μg/mL), and A488 LDL (1 μg/mL) for 1 hour. Plates were washed with ice-cold PBS 3 times, followed by trypsinization and flow cytometric analysis. Apoptosis assays were performed using FITC-labeled Annexin V and 7-amino-actinomycin D (eBiosciences) according to the manufacturer’s protocol. Flow cytometry data was analyzed using FlowJo software.

### Bacterial DNA isolation, 16S rRNA gene sequencing, and microbiome functional analysis.

Experiment design and analysis was performed as previously described (25). To evaluate the alteration in several metabolic pathways in response to microbiome changes after FTX, KEGG and PICRUSt analysis (55) of the stool bacteria of *ApoE^–/–^* mice receiving FTX from either NPX and sham-operated cohorts was performed as described previously (25).

### Filopodia detection and measurement.

FiloQuant plugin (134–136) in the ImageJ Fiji environment was used (Supplemental Figure 9). RAW cells were seeded on 6-well plates in DMEM with 10% FBS at 10^3^ cells/mL and allowed to adhere overnight. Cells were then treated with uPC (10 μg/mL) or control under serum starvation for 24 hours (*n* = 12 wells each treatment). Wells were washed with PBS for 5 min 3 times and then fixed with 4% paraformaldehyde in PBS for 15 minutes. Wells were washed again with PBS 3 times before mounting. Random fields (×20 power) phase contrast pictures were taken and saved in TIF format (*n* = 10 each slide). Filopodia were detected and measured (length) following the published manual (https://imagej.net/_images/d/d0/FiloQuant_Manual_V1.pdf). Images with artifacts were excluded. Each field mean filopodia length (inches) was used for analysis. Slides were coded and blindly evaluated during this procedure.

### F-actin staining.

RAW cells were seeded in a similar way as described to above, on 6-well plates with sterile coverslips at the bottom (*n* = 2 plates [12 slides]/group). After 24 hours exposure to uPC under starvation, cells were washed with PBS and fixed with 4% FPA. F-actin was stained using phalloidin-TRITC (Hello Bio, HB8621) following the manufacturer’s instructions. Images (*n* = 6–8 random images/slide) were taken using Leica TCS SP8 confocal microscope system (100× objectives), under the same exposure time.

### Liquid chromatography–tandem mass spectrometry analysis.

Samples were analyzed by liquid chromatography–tandem mass spectrometry (LC-MS/MS) using a Sciex API 3000 triple quadrupole mass spectrometer equipped with a TurboIonSpray source for PCS and an APCI source for uPC, both of which were operated in negative mode. A chromatographic separation was performed on a Shimadzu Prominence HPLC with a Waters XSelect CSH C18 column 2.1 × 100 mM 3.5 μm. The mobile phase for the analysis of PCS was B: 95/5 acetonitrile/water plus 0.1% formic acid and A: 5/95 acetonitrile/water plus 0.1% formic acid. A gradient profile was utilized starting at 10% B and increasing to 95% with a flow rate of 0.26 mL/min. The SRM transitions for uPC and PCS d7 internal standard were 186.8/107.1 and 193.9/114.1, respectively. The analysis of uPC utilized the same column with acetonitrile and water used as the mobile phase. This analysis was a gradient elution starting at 20% B and increased to 95% B with a flow rate of 0.26 mL/min. The SRM transitions for uPC and PCS d3 internal standard were 107.0/106.3 and 110.1/109.4, respectively.

The sample preparation involved weighing the cecum and adding the appropriate amount of 5% methanol to make a 0.2 g/mL that was then homogenized using a Next Advantage Bullet Blender. A protein precipitation was then performed by adding 200 μl of acetonitrile plus 0.1% formic acid to 50 μl of the homogenate. The samples were vortexed and centrifuged. The supernatant was then analyzed. Standards were prepared from 0.02 to 8 μg/mL in serum for *p*-cresol and 0.02 to 40 μg/mL PCS. Total cecum content of each metabolite was then calculated.

### Statistics.

Mann-Whitney *U* or analysis of variance with Tukey’s correction for multiple comparisons where used when applicable after determination of data distributions and variance. GraphPad Prism software was used for statistical analysis and presentation. *P* values less than 0.05 were considered to be statistically significant. Results are presented in mean ± SD.

### Study approval.

Animal studies were performed in accordance with the guidelines and approval of the University at Buffalo IACUC.

## Author contribution

RY, RJQ, MEM, and LDC conceptualized and designed the study. LDC, MAB, and SA conducted the animal experiments. LDC, AMH, and CMS performed the cell and flow cytometry experiments. RY, CMS, and SCW performed the PCR. RY, DIM, and SA performed the 16S rRNA gene sequencing experiments and analysis. DMR performed the LC-MS/MS experiment. All authors participated in the data analysis, manuscript preparation, and approved the final version.

## Supplementary Material

Supplemental data

Supplemental Table 1

## Version 1 04/29/2021

In-Press Preview

## Version 2 06/08/2021

Electronic publication
