# Supplementary material for: Unconjugated p-cresol activates macrophage macropinocytosis leading to increased LDL uptake
Source: JCI Insight. 2021 Jun 8;6(11):e144410. doi: 10.1172/jci.insight.144410 (PMC8262368; doi:10.1172/jci.insight.144410)
Supplement: Supplemental data [file jciinsight-6-144410-s187.pdf]

Supplementary Figures

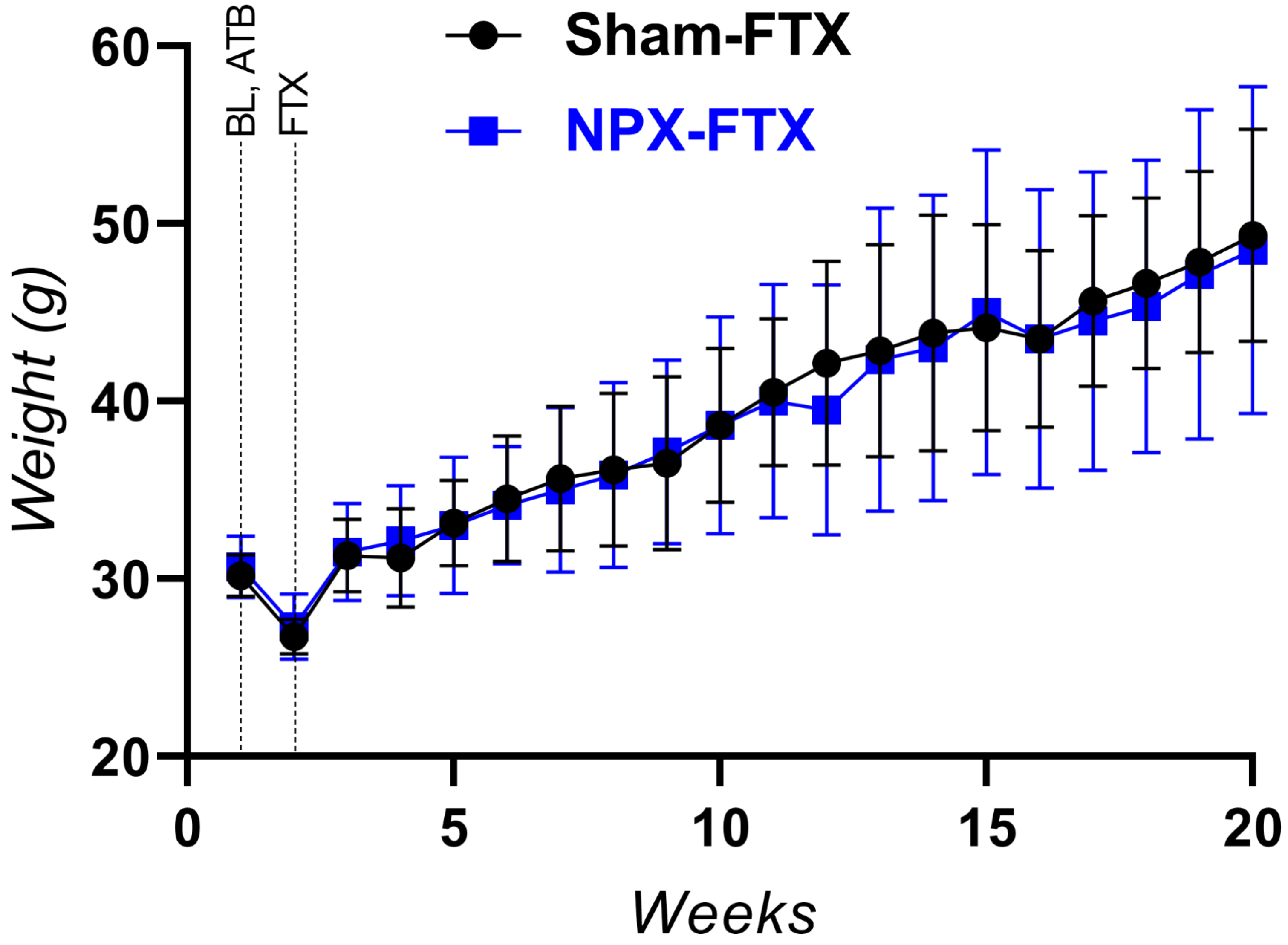

Supplementary Figure 1: *ApoE*$^{-/-}$ mice weight changes after receiving fecal transplantation (FTX). No significant difference in weight was noted between mice receiving FTX from 5/6 nephrectomy (NPX) and Sham operation fecal materials donors.

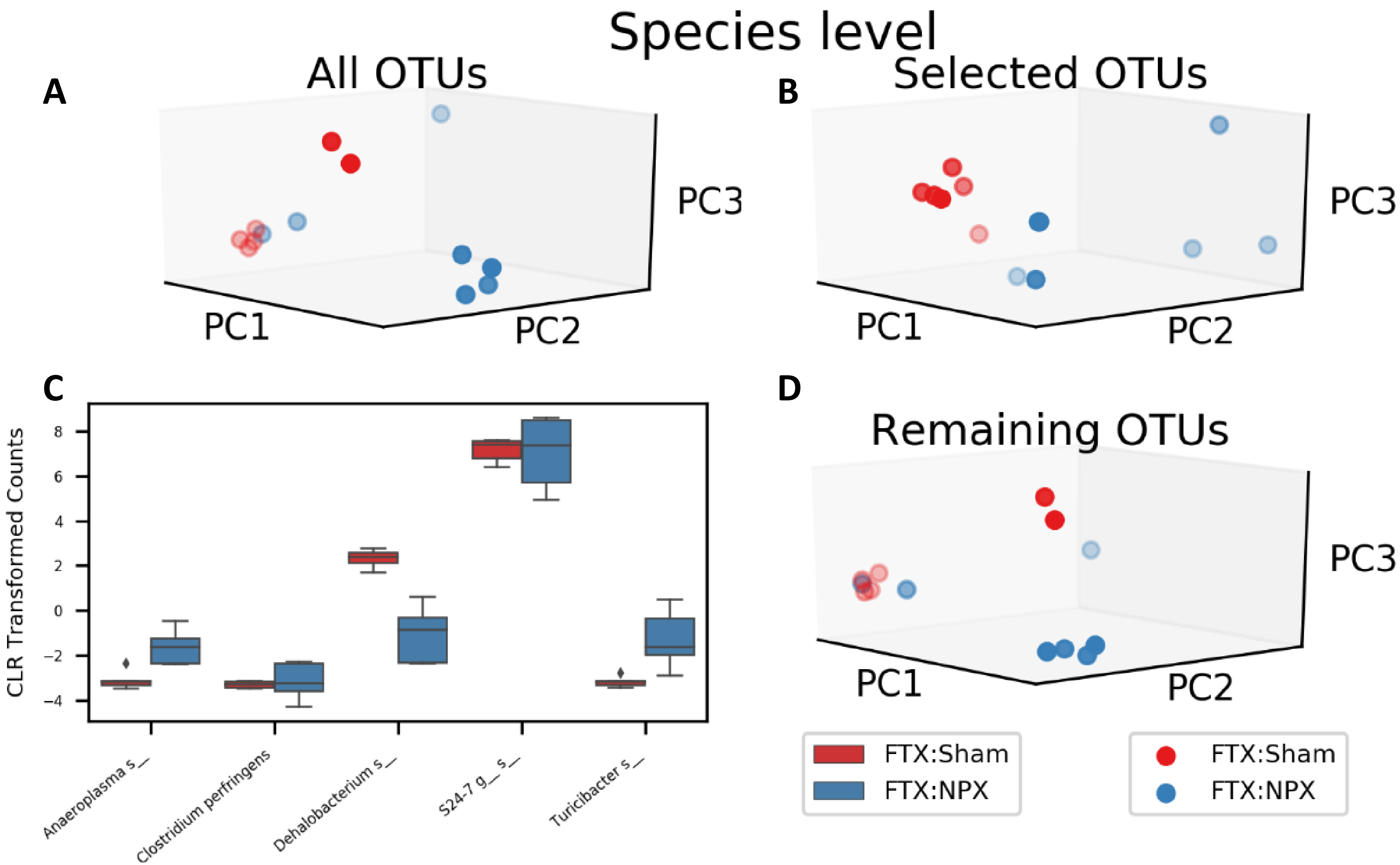

Supplementary Figure 2: 16S sequencing after fecal transplantation (FTX). A clustering of operational taxonomic units (OTUs) on species level was noted in FTX recipients according to the fecal material received (A). While OTUs with statistically different abundance further separated the two groups (B,C), the remaining OTUs were still clustered in response to the FTX donor groups indicating subtle changes in multiple other OTUs (D).

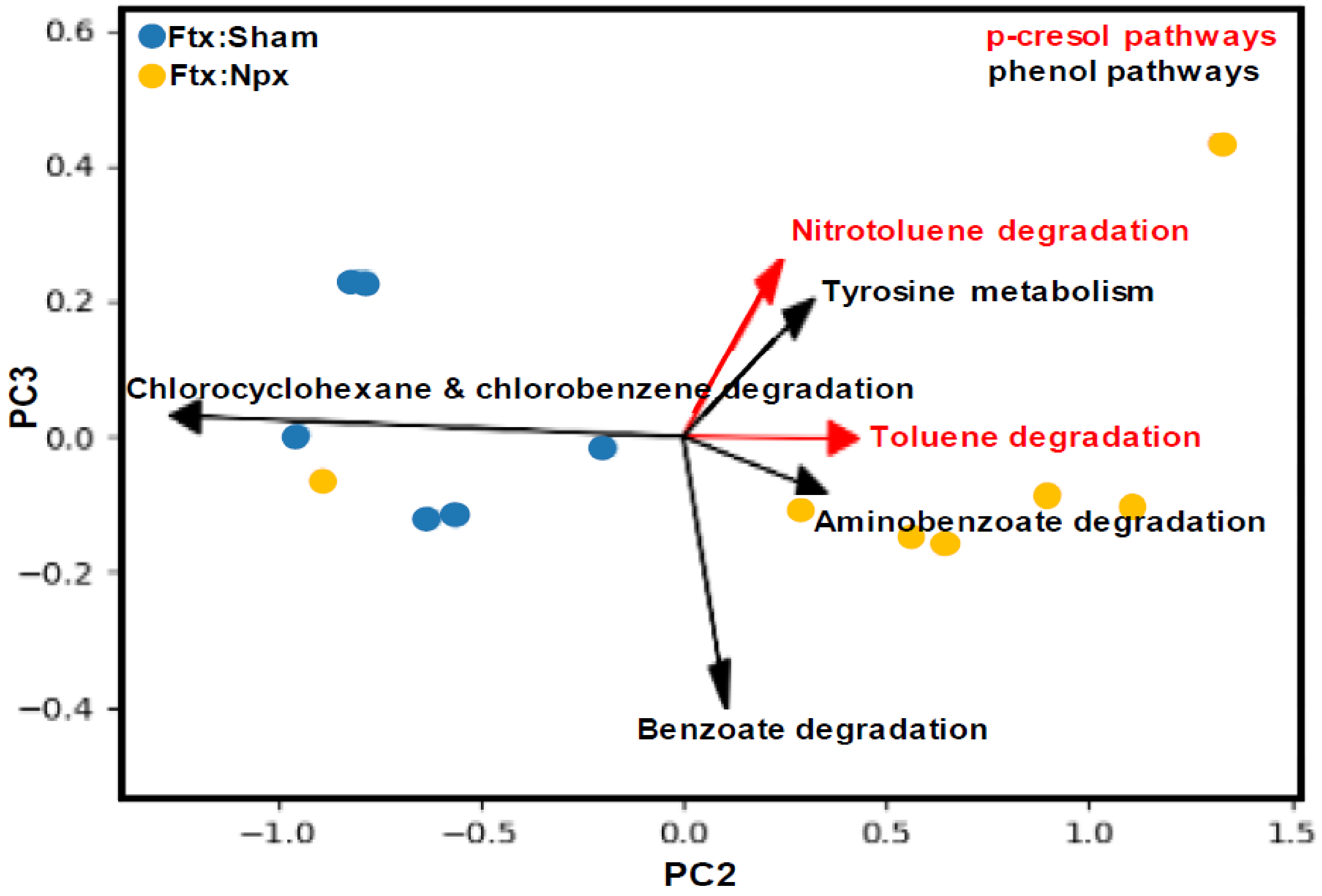

Supplementary Figure 3: PICRUSt functional analysis of stool bacteria after fecal transplantation (FTX). Microbiota functional analysis reveals increased *p*-cresol formation and decreased phenol degradation in mice receiving FTX from 5/6 nephrectomy (NPX) fecal materials donors when compared to those receiving FTX from Sham donors.

Amino Acid Metabolism

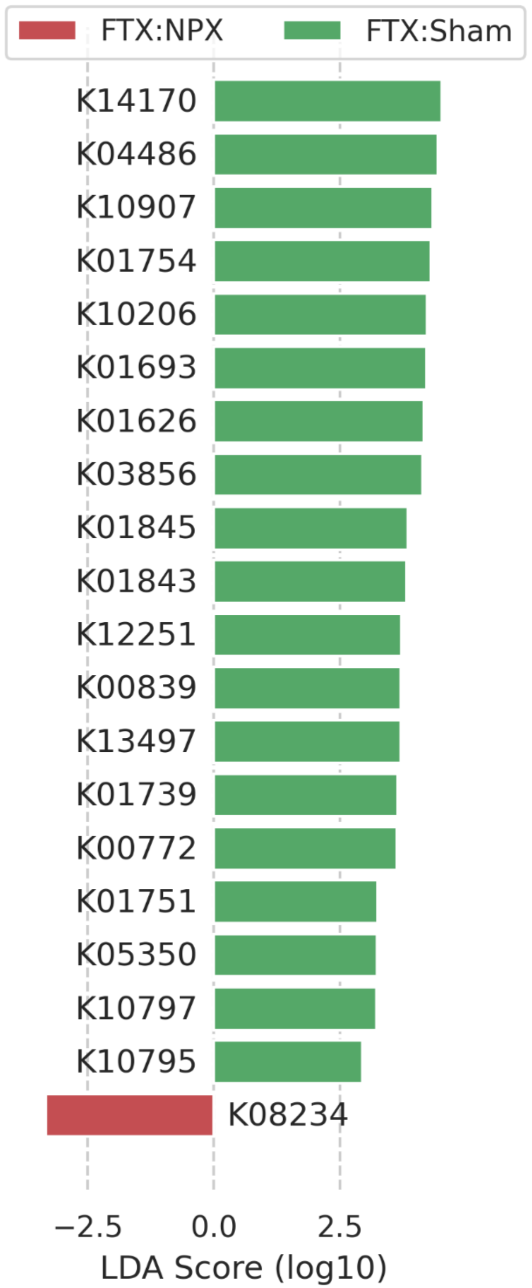

Carbohydrate Metabolism

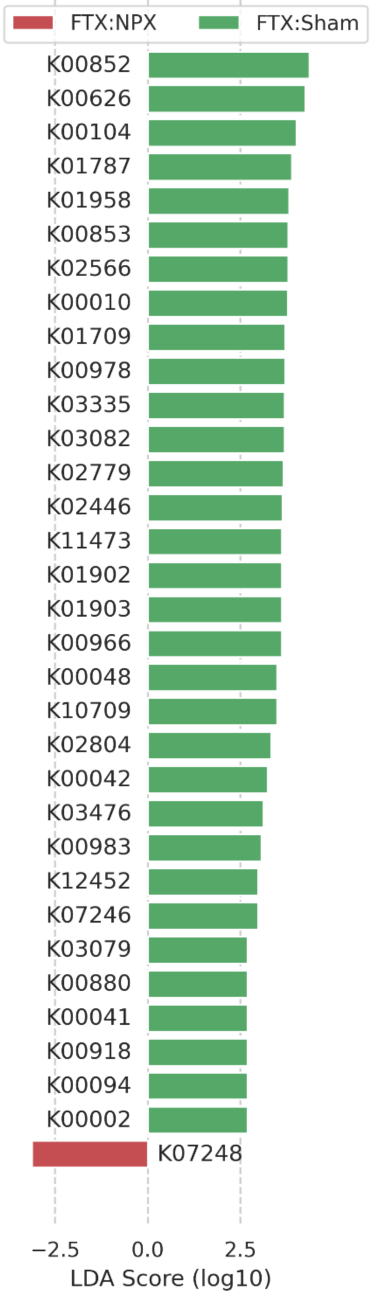

Lipid Metabolism

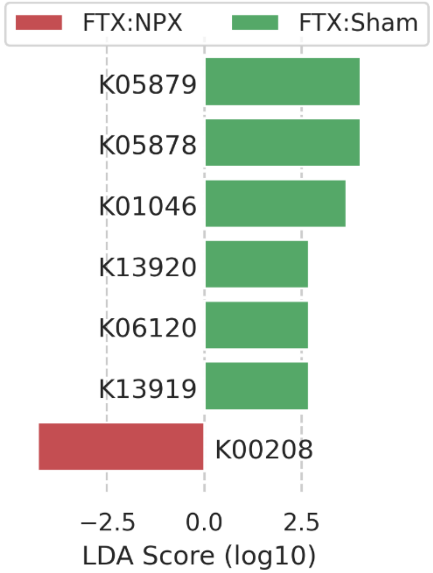

Energy Metabolism

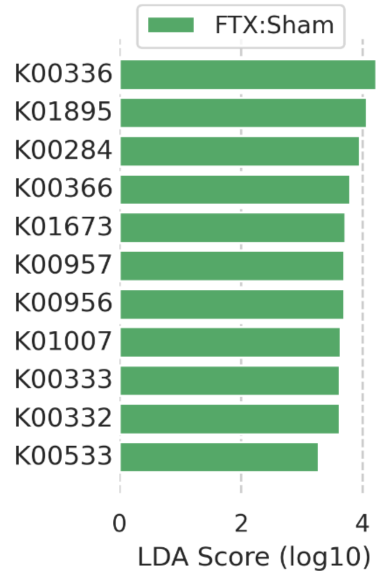

Nucleotide Metabolism

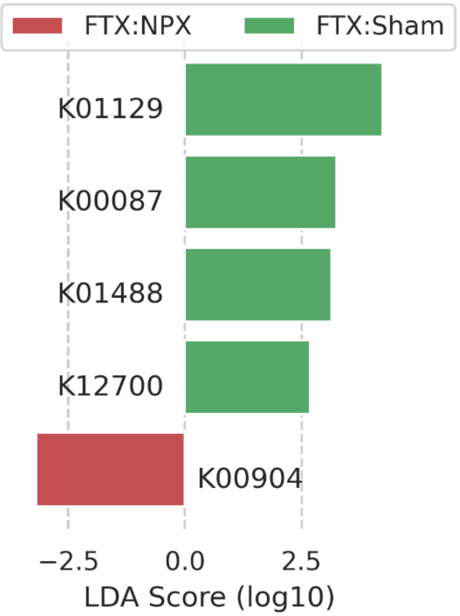

Xenobiotics Biodegradation and Metabolism

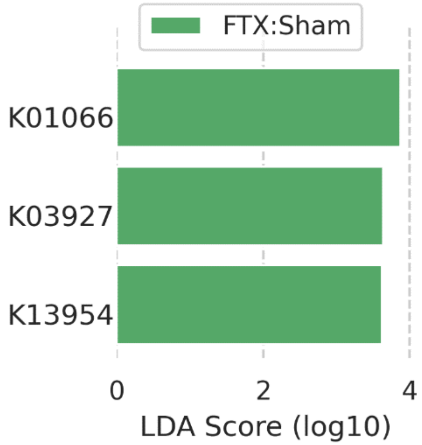

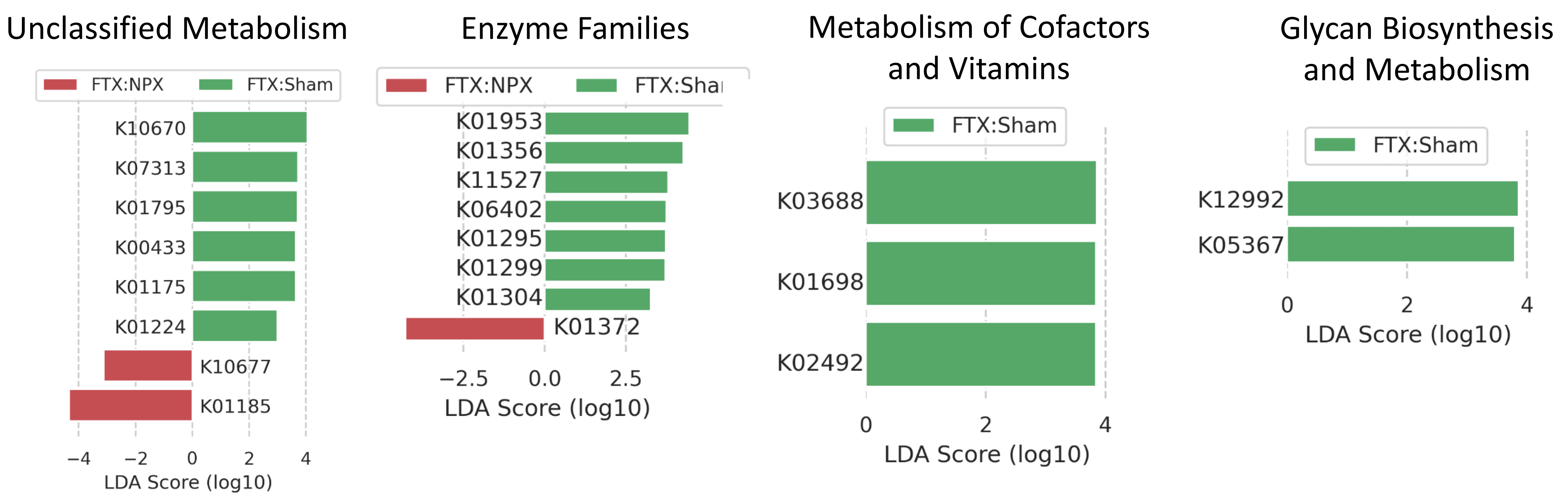

Supplementary Figure 4: PICRUSt based Kegg Orthology (KO) metabolic pathways changes of stool microbiota in response to fecal transplantation (FTX). Amino acid, carbohydrate, lipid, energy, nucleotide, Xenobiotics, cofactors and vitamins, enzyme, and glycan biosynthesis and metabolism differences between mice receiving FTX from from 5/6 nephrectomy (NPX) fecal materials donors and those receiving FTX from Sham donors.

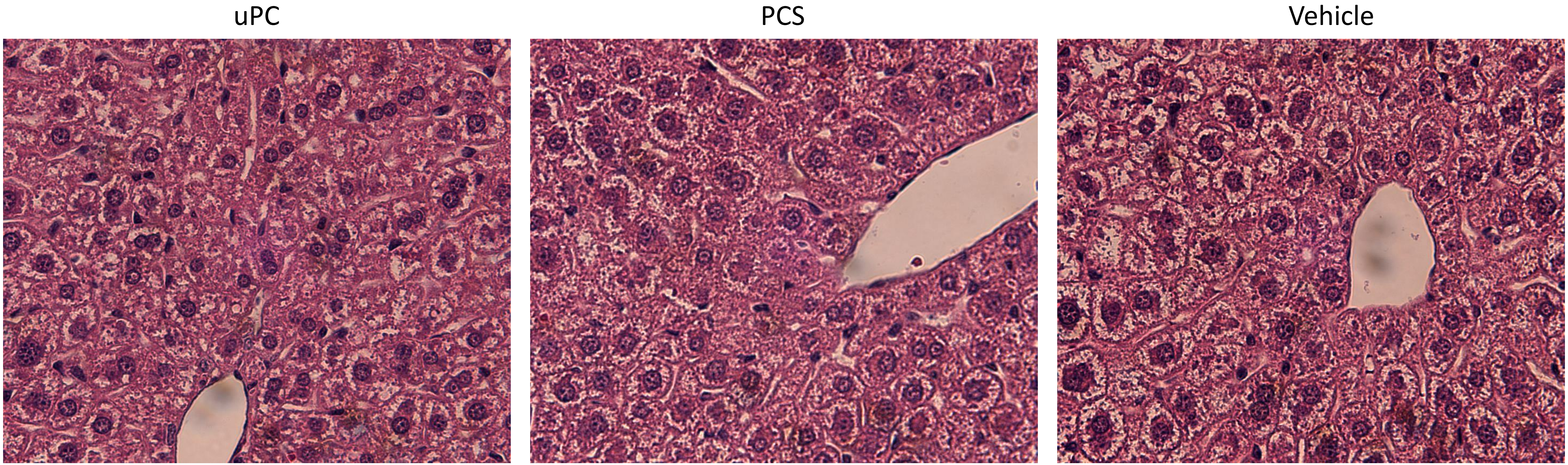

Supplementary Figure 5: Hepatic lipid accumulation in response to unconjugated p-cresol (uPC) and p-cresol sulfate (PCS) in C57BL/6J mice fed standard laboratory chow. No significant lipid accumulation was noted in response to uPC or PCS treatment when mice received standard laboratory chow.

A

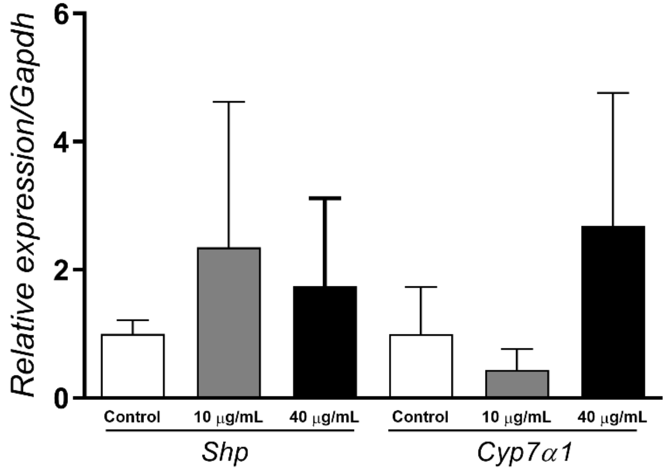

B

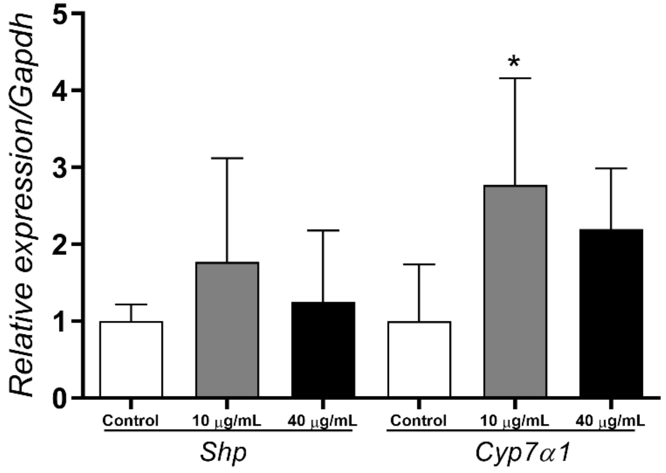

Supplementary Figure 6: Cholesterol efflux gene expression in RAW cells. Reverse cholesterol transport system gene expression analysis in response to exposure to both unconjugated *p*-cresol (uPC, Figure A) and *p*-cresol sulfate (PCS, Figure B). A trend towards decreased expression of *Cyp7a1* in response to 10 µg/mL uPC, along with a significant increase in *Cyp7a1* expression in response to both 10 and 40 10 µg/mL PCS was noted. *n* = 6 each treatment. *p<0.05 (Tukey test).

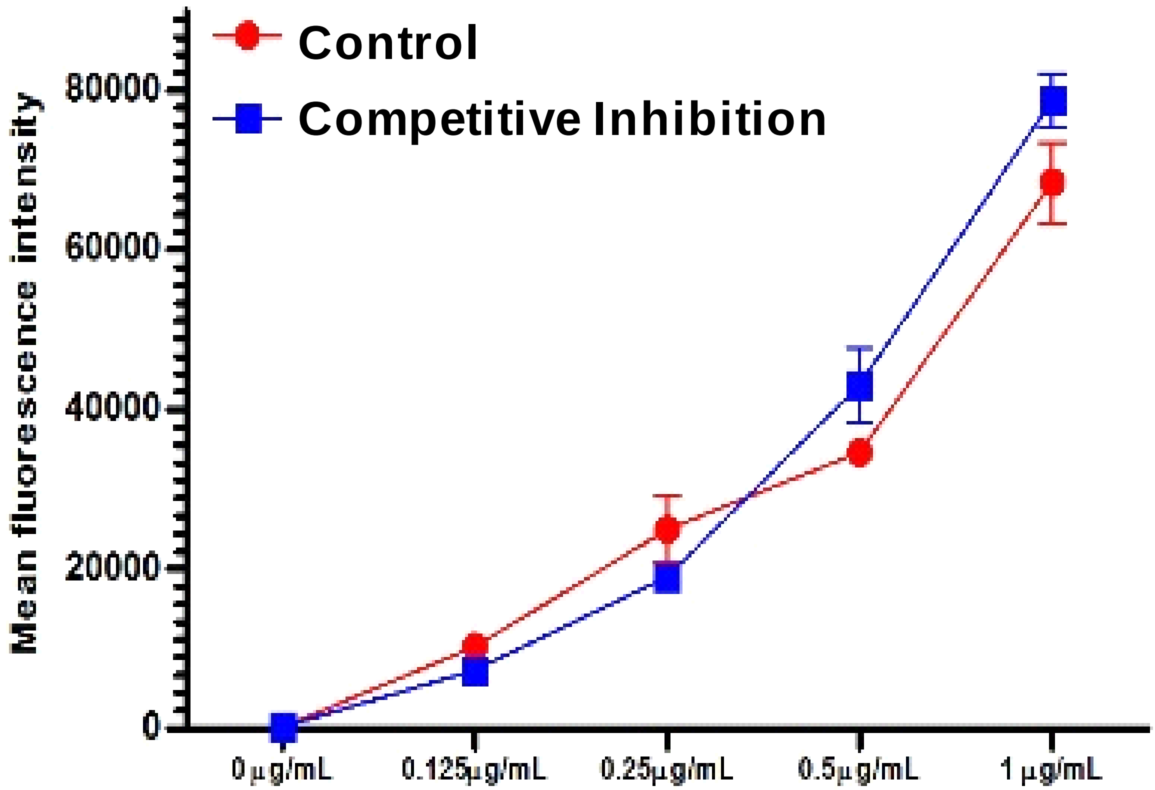

Supplementary Figure 7: Competitive inhibition of receptor mediated endocytosis of Alexa-fluor 488 labeled LDL in RAW cells. Increased A488 LDL (x axis) resulted in increased fluorescence intensity regardless of the presences of 200X unlabeled LDL to inhibit receptor mediated endocytosis confirming that macrophage LDL uptake is not governed by receptor mediated endocytosis. *n* = 3 each treatment/time point.

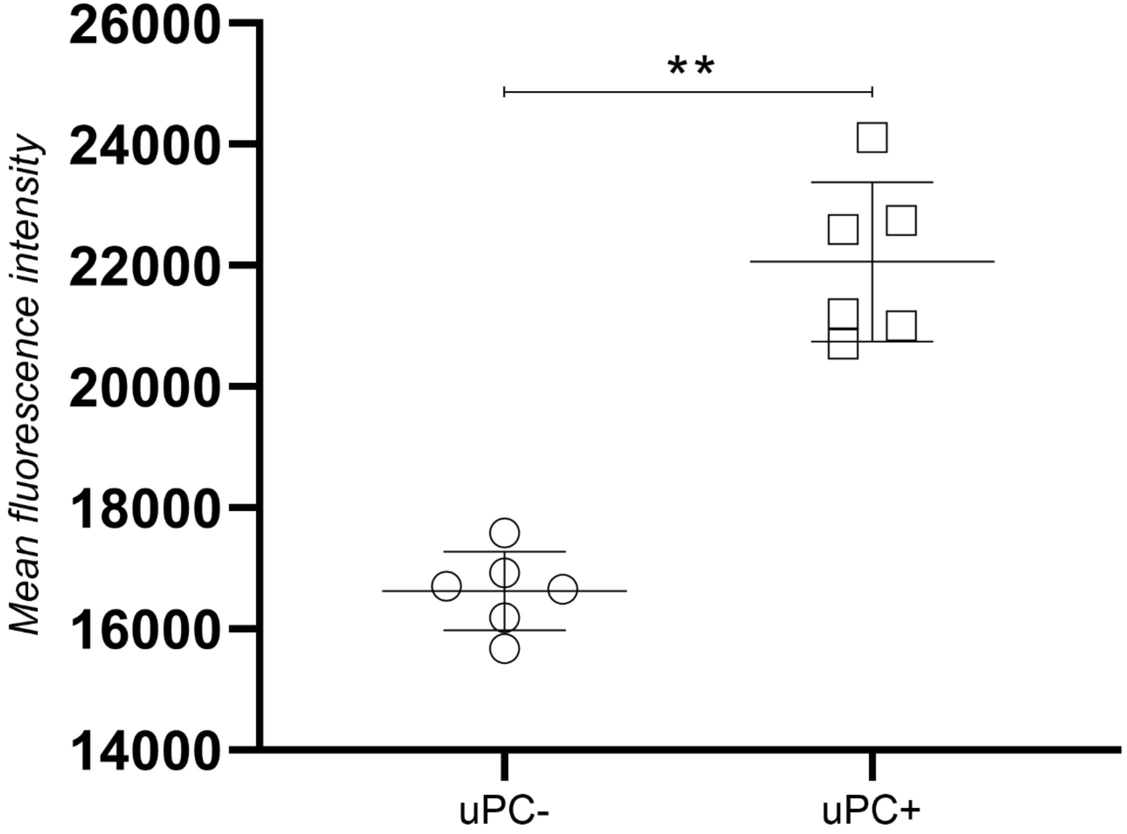

Supplementary Figure 8: A488 LDL uptake in response to 1 µg/mL unconjugated *p*-cresol (uPC) exposure. RAW cells exposed to 1 µg/mL uPC for 24 hours under starvation followed by 1 hour A488 LDL exhibited increased LDL uptake. ***p*<0.01 (Mann-Whitney).

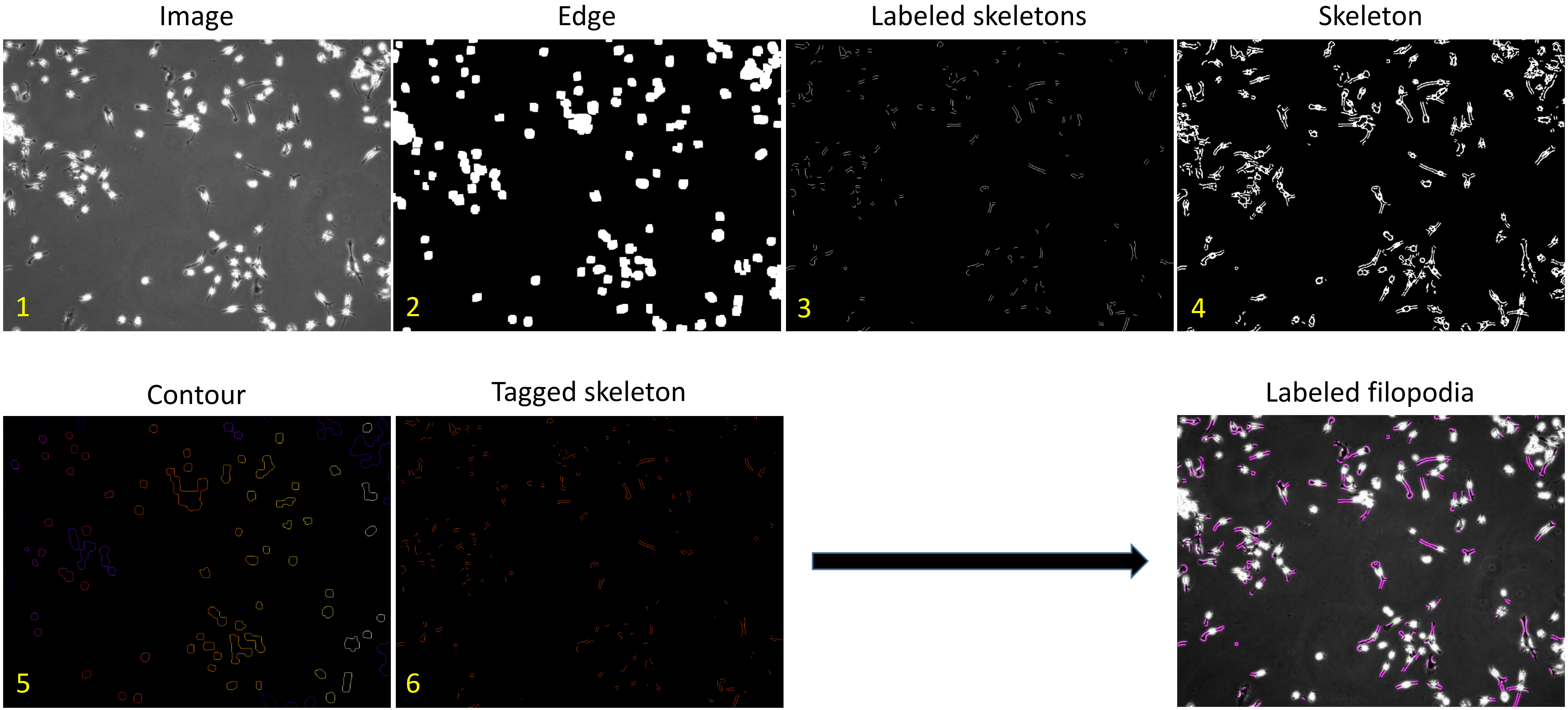

Supplementary Figure 9: Automated filopodia detection using FiloQuant in ImageJ Fiji.

Supplementary Table

Gene	Forward primer	Reverse primer	Product size
Abca1	CAATCCCAGATACCCCTTGC	GGAAGAGGTCCACGATGCTC	81
Abcg1	TTCTGACCTTTCCCCTGGAG	AACAGCACAAAACGCACAGC	190
Abcg5	AATGTGCCTTCAAGGAATCTGAC	TACTCGCCTCAGCAGGACAC	149
Abcg8	CACCTACCAGGTGGACATCG	CAGCCTGAGCTCCCTATGATG	171
Shp	ACCCTCACTGGCTGCAGTTC	CTGGCACATCTGGGTTGAAG	122
Srb1	GCAGCAGGTGCTCAAGAATG	TCCCGGACTACTGGCTTCTG	153
Gapdh	GCCATCAACGACCCCTTCAT	ATGATGACCCGTTTGGCTCC	269
Ldlr	AGGAGTGCAAGACCAACGAG	ACAGGCACTCAGAGCCAATC	87
Rhoa	GCCAAAATGAAGCAGGAGC	ATGAGGCTGCGTTCACAAG	211
Mylip	ACAGGAGCAGACAAGGCATATC	CTTCGCAACGATGCTGTTC	214
Rac1	GTGGGAGACGGAGCTGTTG	GTCAAAGACGGTGGGGATG	90
Nr1H2	GAAGGCGTCCACCATTGAG	TGGCGATAAGCAAGGCATAC	215
Nr1H3	TACAACCGGGAAGACTTTGC	TGCAGAGAAGATGCTGATGG	141
Cdc42	GCGGAGAAGCTGAGGACAAG	GAGTGTATGGCTCTCCACCAATC	221
Cyp7a1	TAAGACGCACCTCGTGATCC	TCAGGGCTCCTGATCATTTG	90

Supplementary Table 2: Real time PCR primers used.
